# Unusual Stroke-Like Presentation of Tumefactive Multiple Sclerosis in a 27-Year-Old Man

**DOI:** 10.7759/cureus.79909

**Published:** 2025-03-02

**Authors:** Mohamed M Abd Elhamid, Hussein W Khudhur, Moustafa A Hemid, Fatema Alshehhi, Ahmed Y Osman

**Affiliations:** 1 Education, Abu Dhabi Health Services Company (SEHA) Sheikh Khalifa Medical City, Abu Dhabi, ARE; 2 Radiology, Abu Dhabi Health Services Company (SEHA) Sheikh Khalifa Medical City, Abu Dhabi, ARE; 3 Internal Medicine, Abu Dhabi Health Services Company (SEHA) Sheikh Khalifa Medical City, Abu Dhabi, ARE

**Keywords:** demyelinating lesions, multiple sclerosis, pseudotumoral demyelinating lesions, stroke mimic, tumefactive demyelination, tumefactive multiple sclerosis

## Abstract

Tumefactive multiple sclerosis (TMS) is a rare subtype of multiple sclerosis with diverse clinical presentations, often leading to delays in diagnosis and treatment initiation. On investigation, it presents with a large (>2 cm) demyelinating mass-like lesion and can be accompanied by ring enhancement, perilesional edema, and mass effect. We report the case of a 27-year-old man who presented with right-sided weakness and numbness. He had a similar presentation one year ago without a definitive diagnosis. Computed tomography (CT) of the brain showed multiple bilateral hypodensities and vasogenic edema. Magnetic resonance imaging (MRI) of the brain revealed multifocal lesions with partial ring enhancement, which prompted further investigations. Laboratory studies and cerebrospinal fluid (CSF) analysis returned negative for infectious and autoimmune etiologies. Spinal MRI identified focal, non-enhancing lesions in the spinal cord, supporting a demyelinating process. The patient was diagnosed with TMS and received five days of IV methylprednisolone, resulting in significant improvement. This case highlights the importance of clinical suspicion of TMS, which can mimic other etiologies. It also shows the role of efficient history-taking in obviating the need for brain biopsy, which is usually done for similar cases exposing the patient to unwarranted adverse effects.

## Introduction

Tumefactive multiple sclerosis (TMS) is an uncommon subtype of multiple sclerosis (MS) that presents with a wide variety of symptoms, ranging from hemiparesis and aphasia to tonic-clonic seizures [1,2]. On investigation, it mimics central nervous system (CNS) neoplasms, making diagnosis challenging [3]. TMS is defined as a demyelinating mass-like lesion larger than 2 cm and can be accompanied by edema, mass effect, and ring enhancement. Due to these radiological features, it closely resembles CNS lymphoma, toxoplasmosis, metastases, brain abscesses, or glioblastoma, complicating diagnosis and often leading to unnecessary interventions that expose the patient to various risks [4,5]. 

TMS accounts for approximately 2.8%-7% of MS cases and has a female predominance, similar to MS. It is more commonly seen in young adults, though cases have also been reported in pediatrics and elderly populations [5]. Studies have shown that TMS typically follows a relapsing-remitting course of disease [6].

We present the case of a young man who initially presented with stroke-like symptoms and whose imaging findings suggested a neoplastic or infectious process. He was later diagnosed with TMS and responded well to treatment. 

## Case presentation

A 27-year-old man presented to the emergency department with progressive right-sided weakness. He reported a history of stroke-like symptoms one year ago in his home country, though the diagnosis and treatment were not established. The current episode began 10 days prior to admission with dizziness and right lower limb weakness, which he initially ignored. Over the following week, the weakness progressed, eventually involving the right upper limb on the day of the presentation. He denied headaches, fever, seizures, facial weakness, or recent infections. He also reported no history of risky sexual behaviors or consumption of undercooked meat or unpasteurized dairy. 

On examination, the patient was afebrile, normoglycemic, and vitally stable. The neurological assessment revealed right-sided hyperreflexia, hypertonia, muscle weakness (3/5) in the right upper limb and right lower limb, hypoesthesia, and loss of proprioception. Cranial nerves, vision, and cerebellar function were intact. He exhibited a slow, unsteady gait due to the weakness.

**Figure 1:**
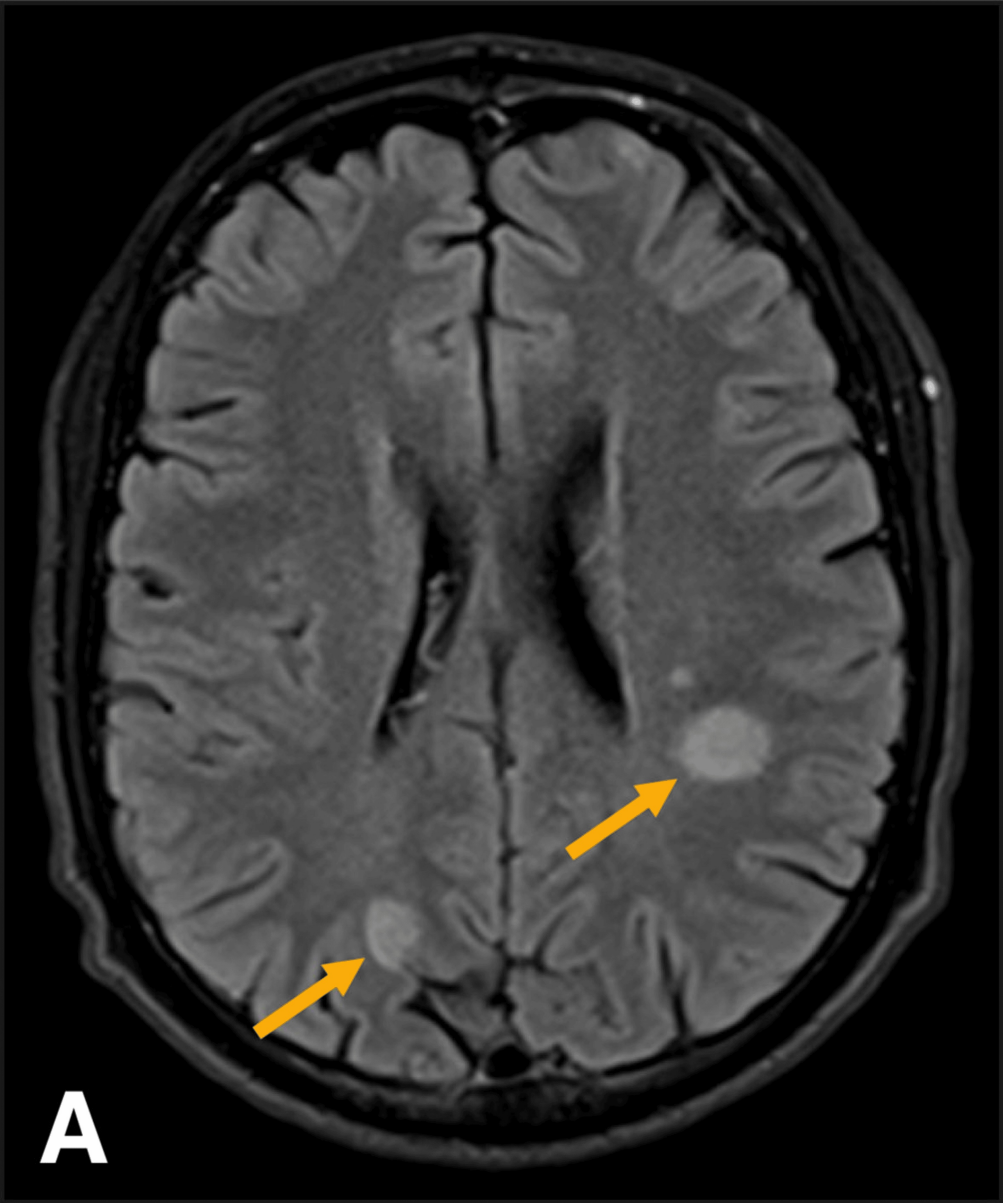
Brain MRI showing multiple focal lesions in both cerebral hemispheres

**Figure 2:**
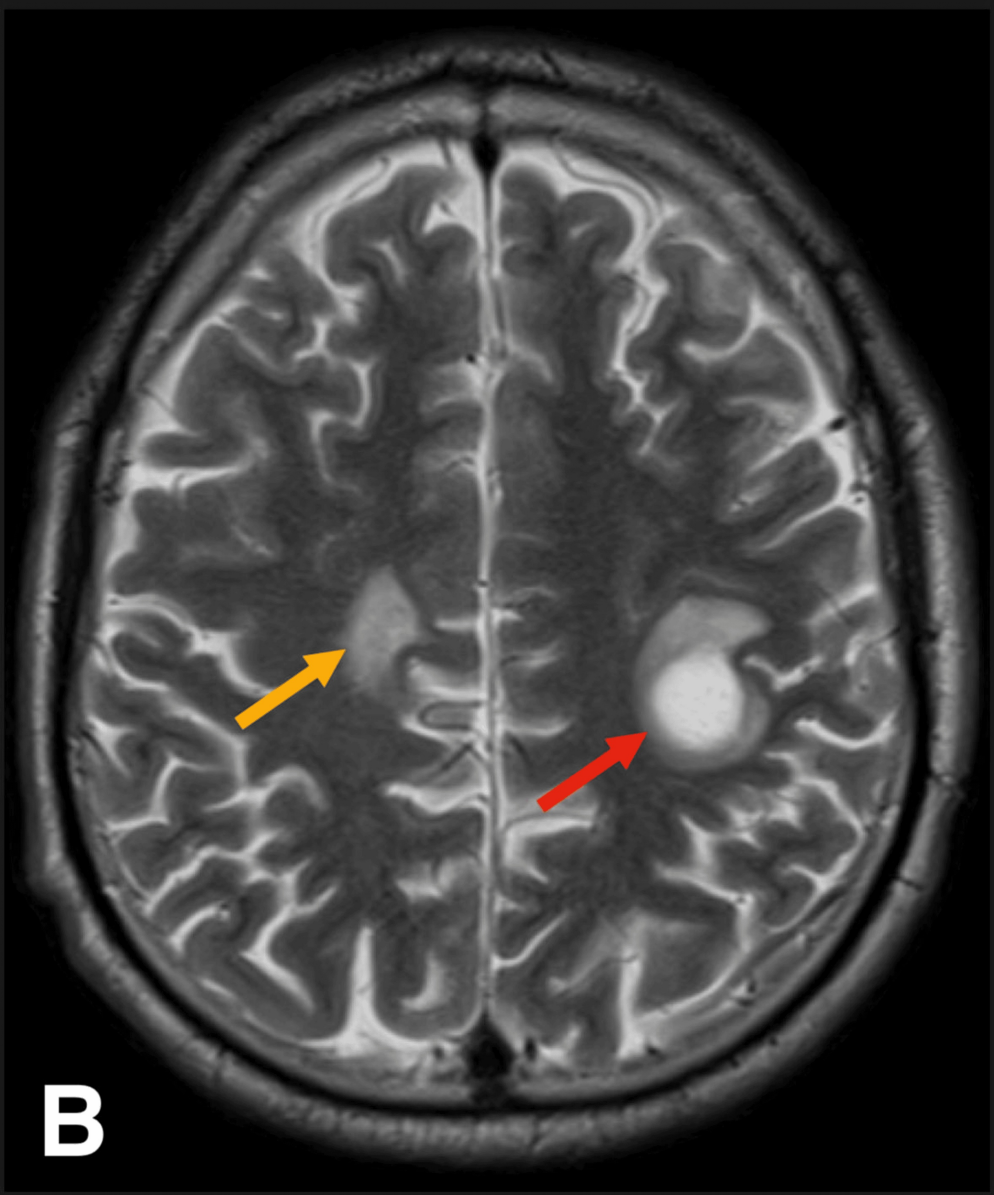
Brain MRI showing multiple focal lesions, including a 2-cm lesion in the left dorsal frontal lobe (red arrow) with partial ring enhancement

Brain CT showed bilateral, asymmetrical supratentorial hypodensities with edema. These findings are nonspecific and may indicate inflammatory, vascular, or neoplastic processes. The delayed onset of presentation and inconclusive CT precluded the activation of the stroke pathway. MRI of the brain (Figure 1 and Figure 2) revealed lesions with partial ring enhancement in the left dorsal frontal lobe (perirolandic region), anterior parietal lobe (parietal operculum), and right parietal lobe (precuneus). Additionally, a lesion without enhancement in the superior frontal gyrus with some cortical encephalomalacia suggested a similar episode with residual lesions in the past.

The near-complete partial ring enhancement raised the possibility of multiple tumefactive demyelinating lesions. The partial ring enhancement is a distinguishing feature that helps differentiate TMS from neoplastic and infectious lesions, which typically exhibit complete ring enhancement. Additionally, the presence of non-enhancing lesions suggests chronic demyelination, further supporting a demyelinating disorder rather than a space-occupying lesion. However, the presence of multiple tumefactive demyelinating lesions without additional white matter changes is unusual. As a result, inflammatory processes, particularly parasitic infections such as toxoplasmosis or neurocysticercosis, were considered. Other possibilities included granulomatous infections such as tuberculosis, fungal infections, and neoplastic conditions like metastases and lymphoma, though these were deemed less likely.

Laboratory tests included a complete blood count with differential and electrolytes that were normal. Serological tests were negative for infectious and autoimmune etiologies (Table 1), including human immunodeficiency virus, tuberculosis, echinococcosis, cysticercosis, and brucellosis. The test results indicated positive toxoplasmosis immunoglobulin G (IgG) and negative immunoglobulin M (IgM), which suggests a past infection rather than an active one. Blood tested negative for monoclonal components, and immunoglobulin levels were normal.

**Table 1:** Blood test results

Test name	Result	Reference value	Interpretation
HIV Ag/Ab screen	Negative	Negative	No HIV infection detected
Antineutrophil cytoplasmic antibodies (ANCAs: PR3, MPO)	Negative	<2 RU/mL	No evidence of ANCA-associated vasculitis
C-reactive protein (CRP)	0.50 mg/L	<5 mg/L	No significant inflammation
C3 complement	1.23 g/L	0.9-1.8 g/L	Normal levels
C4 complement	0.24 g/L	0.1-0.4 g/L	Normal levels
Cyclic citrullinated peptide (CCP)	1.20 U/mL	Negative (<20 U/mL)	No evidence of rheumatoid arthritis
Rheumatoid factor (RF) quantitative	<3.6 CU	Negative (<14 CU)	No evidence of rheumatoid arthritis
Extractable nuclear antigen (ENA) screen	Negative	Negative	No autoimmune antibodies detected
Echinococcus antibodies (Echino Ab)	<1:160	Negative	No evidence of echinococcosis
Toxoplasma IgG	Positive	>3.40 IU/mL	Past exposure to toxoplasmosis
Toxoplasma IgM	Negative	Negative	No active infection
QuantiFERON-TB	Negative	Negative	No tuberculosis infection
*Brucella *IgG	Negative	<2.00 RU/mL	No *Brucella *exposure
*Brucella *IgM	0.04	Negative	No active *Brucella *infection
Cysticercosis (*Taenia solium*) IgG	Negative	Negative	No evidence of cysticercosis

Cerebrospinal fluid (CSF) analysis was negative for mycological and bacteriological cultures and the absence of oligoclonal bands. The CSF Venereal Disease Research Laboratory test was negative. CSF analysis of Cysticercosis, Neuromyelitis Optica (NMO)/Aquaporin-4-IgG Fluorescence-Activated Cell Sorting (FACS), and adenosine deaminase all returned negative.

**Table 2:** Cerebrospinal fluid (CSF) test results VDRL: Venereal Disease Research Laboratory.

Test Name	Result	Reference value	Interpretation
Oligoclonal banding (CSF)	1 band (CSF)	<2 bands (negative)	No significant intrathecal IgG synthesis (not suggestive of multiple sclerosis)
Oligoclonal banding (serum)	0 band	Negative	No abnormal bands detected
VDRL (CSF)	Negative	Negative	No evidence of neurosyphilis
Adenosine deaminase (CSF)	<1 U/L	0-9 U/L	No evidence of tuberculous meningitis
CH50 (total hemolytic complement)	55 U/mL	>32 U/mL	Normal complement function
NMO/AQP4 IgG (CSF)	Negative	Negative	No evidence of neuromyelitis optica (NMO)

**Figure 3:**
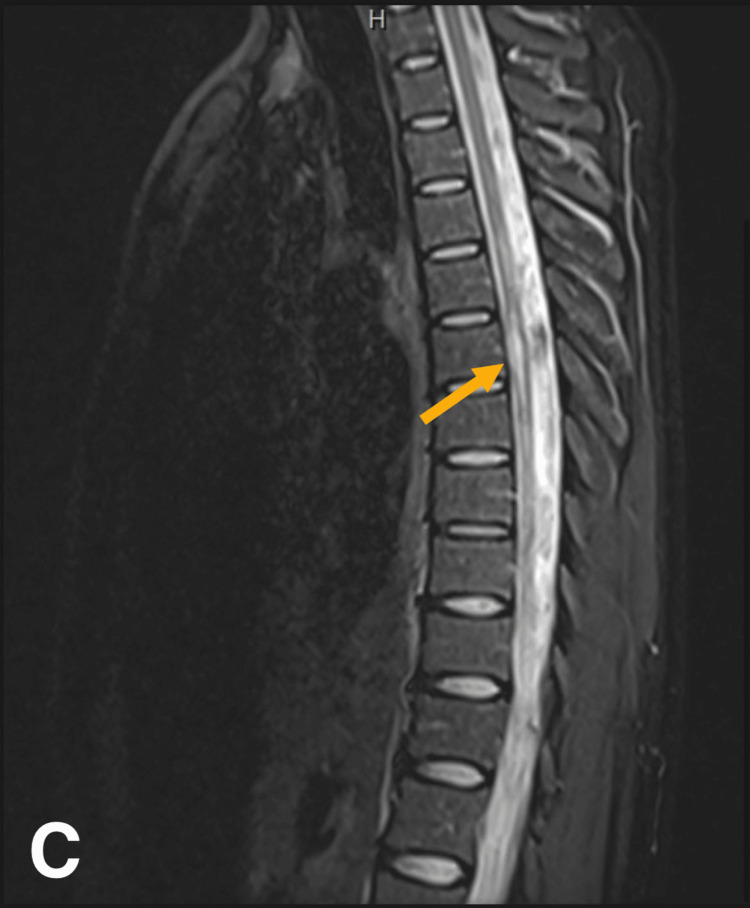
Spinal MRI showing short segmental enhancing lesion at T6-T8 (arrow) correlating with the T7 sensory level

The neurology team was consulted, and on examination, a sensory level at T7 was revealed. Spinal MRI (Figure 3) revealed a focal non-enhancing lesion in the spinal cord at C3, as well as short segmental enhancing lesions at T6-T8 and T11-T12. These findings were highly suggestive of a demyelinating process, most likely TMS. 

The patient was diagnosed with TMS and received a seven-day course of IV methylprednisolone at a dosage of 1,000 mg per day. Throughout the treatment, his condition improved significantly. His muscle strength increased from grade three to grade four, the numbness decreased, and he gradually regained the ability to walk without support. Upon discharge, he was transitioned to oral prednisolone with a tapering regimen and scheduled for a follow-up appointment at the internal medicine clinic in one week. At follow-up, he had a complete resolution of symptoms, except for residual numbness on the right side. He was subsequently scheduled for a neurology appointment to discuss long-term treatment plans with disease-modifying therapy.

## Discussion

TMS has been classically defined as acute large >2 cm, tumor-like demyelinating lesions in the CNS that is usually accompanied by perilesional edema, mass effect, and, most importantly, ring enhancement [3]. TMS lesions can mimic a variety of CNS lesions, such as toxoplasmosis, CNS lymphoma, and primary brain tumors like glioblastoma [1]. Patients with MS can have a TMS as their initial presentation [3].

TMS has a wide variety of presentations depending on the site of the lesions, which makes it difficult to diagnose. According to previous case reports and case series, it ranges from headaches to cognitive abnormalities, confusion, aphasia, foot drop, tonic-clonic seizures, and hemiparesis [7]. As seen in our case, the presenting features of sudden onset of right-sided weakness and paresthesia, along with the inconclusive findings on CT, made the diagnosis challenging.

As reported in previous case series, brain biopsy may be required to diagnose TMS, which can lead to adverse events given the invasive nature of the procedure [7]. However, a detailed history-taking might reveal previous neurological events that may obviate the need for a brain biopsy. In our case, the presence of a previous history of a similar event reduced the necessity for a brain biopsy.

TMS responds well to MS medications, corticosteroids in the flare-ups, and disease-modifying drugs. However, due to the diagnostic challenge TMS poses to physicians, treatment is often delayed, which adversely impacts the prognosis [2]. In our case, the patient could have benefited from the early initiation of disease-modifying drugs after his initial presentation.

Most of the patients (90%) who present with a tumefactive demyelinating lesion will go on to develop MS [8]. Disease-modifying therapies (usually immunotherapies) can help decrease the frequency of relapsing MS attacks in these patients [9]. Glatiramer acetate is a synthetic protein that modifies helper T-cell activity and helps reduce inflammation [10]. Glatiramer acetate was approved by the Food and Drug Administration after it was shown to reduce the relapse rate of MS by 33% and preserve ambulation in approximately 80% of patients after 20 years of follow-up [11]. Glatiramer acetate is the only disease-modifying agent that seems to be associated with subsequent lesion size regression [12,13].

## Conclusions

TMS is a rare variant of MS that presents a wide variety of symptoms. Its nonspecific presentation and radiological similarity to other conditions delay diagnosis and treatment. Our case highlights the importance of early clinical suspicion and thorough history-taking in avoiding unnecessary invasive interventions, such as brain biopsy, which is often used to diagnose TMS. Given that TMS predominantly follows a relapsing-remitting course, physicians should remain aware of its ambiguous presentation. Prompt recognition enables the timely initiation of disease-modifying therapies, which can help prevent relapses and improve patient outcomes. Future research should focus on developing standardized diagnostic guidelines for TMS to distinguish it from mimics and expedite treatment initiation.
